# News and Miscellany

**Published:** 1900-03

**Authors:** 


### News and Miscellany.

**The Journal Office** was recently gladdened by a visit from Mr. Mellier of the Mellier Drug Co., St. Louis.

**Dr. E. S. Pettijohn** of the Alma Sanitarium has turned over that institution to Dr. Geo. F. Butler, of Chicago and gone on a tour of Europe, "to rest and study."

**Surgical Instruments,** second hand, if in good order, wanted by a subscriber of the "Red Back." Send list and lowest cash price. Dr. Oliver, care TEXAS MEDICAL JOURNAL, Austin, Texas.

**Don't forget** that the Texas State Medical Association will meet at Waco, Tuesday April 24, prox. The program and list of section officers, etc. will appear in our April number before date of meeting.

**Hello, Doctor.**—Make a note of this and act while it is hot. If you are sending the exchange copy of your journal to Prof. Smith at Galveston, late associate editor of the "Red Back", please change address and send to the JOURNAL at Austin.—D.

**Died in Broctor, Texas,** Feb. 2 (ult.), Mrs. Rebecca J. Eargle, wife of Dr. J. J. Eargle of that city. Mrs. Eargle was Miss Burton, a native of Bosque County, Texas. She was in her fortieth year, much beloved for her qualities of head and heart. Her death is regretted by a large circle of attached friends. The JOURNAL extendsit s sympathy to Dr. Eargle and the bereaved children.

**Dr. Bat Smith** of Wharton, Texas, who, in the fifties, was a student under the great N. O. Pathologist Schmidt and who is a yellow fever expert, we are gratified to note, has been appointed chief surgeon (U. S. A.) of the yellow fever hospitals in Santiago, Cuba. Dr. Smith was surgeon in the Texas Contingent U. S. V. in the late war with Spain and served in Cuba. The JOURNAL has been favored by Dr. Smith with a paper on yellow fever, and it will appear in our next issue.

**The report** has been circulated that the New Orleans Polyclinic has been suspended on account of smallpox in New Orleans.

This statement is absolutely false. The Polyclinic has had a continuous course since November 20, and will continue until May 12. The smallpox situation in. New Orleans has at no time justified any apprehension on the part of the students attending the Polyclinic or on the part of those who might wish to do so.

Isadore Dyer, M. D.,
Secretary, New Orleans Polyclinic.

---

**The New Orleans Polyclinic,** thirteenth annual session, opens November 30, 1899; closes May 10, 1900. Every inducement in clinical facilities for those attending. The specialties are fully taught. Further information, New Orleans Polyclinic, New Orleana, La.

---

**For Sale.**—Eye, Ear, Nose and Throat Practice, with books, instruments and office furniture, in a growing town of 12,000 inhabitants. No other specialist within a hundred miles. A good opportunity for a specialist or a physician who would like to do both special and general practice. Address "Doctor," in care Texas Medical Journal.

---

**The Called Session of the Legislature** refused to raise the salaries of the asylum superintendents in accordance with the Governor's request. The reason as given us by the members was that the Governor did not ask that the salaries of the assistant physicians be raised also. By the "abrogation" of the long established custom of boarding the medical officers of these institutions the salaries of superintendents are $2000. The salaries of assistant superintendents, first and second, are $1250 a year. Had the Governor's request that the superintendents' salary been respected and the assistants' left at $1250, it would have been unjust. At this date Superintendent Wilson has not been relieved. No one wants the position.

---

*Editor Texas Medical Journal:*

The congressional conference of representatives of all state medical societies which was to have been held at Washington in February (ult.) has been deferred until *about* the 15th, inst. The cause of delay, I am advised, was the neglect of state associations to appoint delegates who would attend. Dr. H. L. E. Johnson, of Washington City is chairman of the committee from the National Medical Association, who has charge of the matter. I had this advice from Dr. Tyner a few days ago.

R. H. HARRISON, SR.

Dr. Harrison, Columbus, Texas, and Dr. Tyner, of Washington City were the delegates from Texas State Medical Association. The conference was to revise the organization of army and navy medical departments and to endeavor to secure some national santary and medical legislation.

---

**It has been announced** that the Board of trustees of the University of Pennsylvania has finally secured entire control of the University Medical Magazine, and that in the future it will be published under the auspices and with the support of the University. Heretofore the magazine has been in the hands of a stock company, over which the University had no control.

Owing to the delay attending the transfer of the property, and also owing to the time required for a thorough reorganization, it will be impossible to issue numbers for January or February; the first number of the year will appear in March.

It is proposed that the Magazine shall hereafter be the organ of the Medical Department, and that it shall be creditable to that department. In every way its readers will be kept fully informed as to the work that is being done in the Clinic, the Laboratory, and the Hospital, by the members of the Teaching and Hospital Staff. Contributions upon subjects allied to medicine will be secured, as well, from those engaged in such work at the University.

The liberal policy adopted by the management will enable the Magazine in the future to obtain at all times the most desirable material for publication. Dr. Charles H. Frazier has recently been appointed editor and will at once assume the duties of his office.

